# Immunization with neural derived peptides plus scar removal induces a permissive microenvironment, and improves locomotor recovery after chronic spinal cord injury

**DOI:** 10.1186/s12868-016-0331-2

**Published:** 2017-01-05

**Authors:** Roxana Rodríguez-Barrera, Adrián Flores-Romero, Ana María Fernández-Presas, Elisa García-Vences, Raúl Silva-García, Mina Konigsberg, Liliana Blancas-Espinoza, Vinnitsa Buzoianu-Anguiano, Karla Soria-Zavala, Paola Suárez-Meade, Antonio Ibarra

**Affiliations:** 1 Centro de Investigación en Ciencias de la Salud (CICSA), Universidad Anáhuac México Campus Norte, Huixquilucan, Estado de México Mexico; 2 Facultad de Ciencias de la Salud, Universidad Anáhuac México Campus Norte, Huixquilucan, Estado de México Mexico; 3 Centro de Investigación del Proyecto CAMINA A.C., Ciudad de México, Mexico; 4 Facultad de Medicina, UNAM, Ciudad de México, Mexico; 5 Hospital de Pediatría CMN Siglo XXI, Ciudad de México, Mexico; 6 UIMEN, CMN Siglo XXI, Ciudad de México, Mexico; 7 Lab. Bioenergética y Envejecimiento Celular, UAMI, Ciudad de México, Mexico; 8 Posgrado en Biología Experimental, UAMI, Ciudad de México, Mexico

**Keywords:** A91, Immunomodulation, Neural antigens, Paraplegia, Protective autoimmunity, Regeneration

## Abstract

**Background:**

Immunization with neural derived peptides (INDP) as well as scar removal—separately—have shown to induce morphological and functional improvement after spinal cord injury (SCI). In the present study, we compared the effect of INDP alone versus INDP with scar removal on motor recovery, regeneration-associated and cytokine gene expression, and axonal regeneration after chronic SCI. Scar removal was conducted through a single incision with a double-bladed scalpel along the stump, and scar renewal was halted by adding α,α′-dipyridyl.

**Results:**

During the chronic injury stage, two experiments were undertaken. The first experiment was aimed at testing the therapeutic effect of INDP combined with scar removal. Sixty days after therapeutic intervention, the expression of genes encoding for TNFα, IFNγ, IL4, TGFβ, BDNF, IGF1, and GAP43 was evaluated at the site of injury. Tyrosine hydroxylase and 5-hydroxytryptamine positive fibers were also studied. Locomotor evaluations showed a significant recovery in the group treated with scar removal + INDP. Moreover; this group presented a significant increase in IL4, TGFβ, BDNF, IGF1, and GAP43 expression, but a decrease of TNFα and IFNγ. Also, the spinal cord of animals receiving both treatments presented a significant increase of serotonergic and catecholaminergic fibers as compared to other the groups. The second experiment compared the results of the combined approach versus INDP alone. Rats receiving INDP likewise showed improved motor recovery, although on a lesser scale than those who received the combined treatment. An increase in inflammation and regeneration-associated gene expression, as well as in the percentage of serotonergic and catecholaminergic fibers was observed in INDP-treated rats to a lesser degree than those in the combined therapy group.

**Conclusions:**

These findings suggest that INDP, both alone and in combination with scar removal, could modify the non-permissive microenvironment prevailing at the chronic phase of SCI, providing the opportunity of improving motor recovery.

## Background

After SCI, numerous anatomical and physiological self-destructing mechanisms are triggered. These events induce a discontinuity in the spinal cord (SC) parenchyma [1–3]. One of these events is the inflammatory response, a phenomenon that could exert beneficial effects after SCI [4]. Recent studies have demonstrated that modulation of the immune response confers protective and reparative effects after central nervous system (CNS) injury [5, 6]. This phenomenon—termed protective autoimmunity (PA), is a novel therapeutic paradigm that has been used to promote neuroprotection and neural restoration.

This particular strategy is achieved by immunizing with neural-derived peptides (INDP) such as A91, a peptide derived from the 87-99 immunogenic sequence of amino acids that give structure to the myelin basic protein (MBP). Activation of T-Lymphocytes by A91-peptide induces an anti-inflammatory Th2 response that allows microglia to differentiate into an M2 phenotype. The resulting microenvironment after immunization is characterized by a low production of free radicals and several neuroprotective mechanisms [7, 8]. The therapeutic effect of PA has already been reported when INDP is performed immediately after SCI; however, there is no published data describing the effect of this strategy when administered during the chronic phase of injury.

In order to achieve the beneficial effects of this therapy, it is important to consider the prevailing environment at chronic stages of injury. The glial scar formation—that acts as a physical barrier—is one of the main obstacles to allow the action of this therapeutic intervention. Another important feature of the chronic phase of injury is the lack of molecules that were activated—either as protective or restorative promoters—throughout the acute phase of injury [4]. Chronic SCI is considered a period of stability and low activity at the site of injury, followed by a progressive decline in the neurological function of injured individuals [9].

Under these conditions, scar removal could help to allow regenerating axons to grow across the site of injury, and re-establish the characteristic conditions of the acute phase of the lesion (e.g. neurotrophic factors and cytokine release). The renewed microenvironment could provide the conditions for PA to exert its beneficial actions; especially those related to neural restoration [10]. Taking this approach into consideration, our group developed a reproducible surgical procedure that allows the elimination of the glial scar without causing significant collateral neurological damage. In the first step of this work, we explored whether INDP in combination with scar removal provides an appropriate microenvironment to promote neural restoration. During this preliminary study, we evaluated locomotor recovery, regeneration-associated and cytokine gene expression, as well as the number of regenerating axons, in a model of chronic SCI. The second step compared whether the results of the combined therapy provided better results when compared to INDP alone.

## Methods

### Experimental design

Sample size for this experiment was calculated using an alpha of 0.05 and beta of 0.20. Experiments were performed 60 days after SCI, with subsequent analyses carried out over the two following months. The first experiment consisted of 27 SCI rats randomly distributed in the following three groups (GraphPad QuickCalcs: http://www.graphpad.com/quickcalcs/): (1) sham operated rats (SC is exposed but scar tissue is not removed) immunized with PBS (n = 9); (2) rats with scar removal alone (n = 9); (3) rats with scar removal + INDP (n = 9). The second experiment was composed of 24 SCI rats randomly allocated into three groups: (1) sham operated rats immunized with PBS (n = 8); (2) rats with scar removal + INDP (n = 8); (3) rats with INDP but without scar removal (n = 8). Basal statistical analysis of weight, age, and Basso, Beattie and Bresnahan (BBB) score yielded no statistical significance between experimental groups.

Once animals were allocated into the groups, a blinded surgeon performed the corresponding intervention. After intervention, motor recovery was weekly evaluated for a period of 60 days. At the end of each experiment, rats were euthanized, and the SC was analyzed for the expression of inflammation-related genes. Additionally, we determined the expression of some regeneration-associated genes and the number of regenerating axons.

### Ethics statement

All animals were handled according to NIH guidelines for the management of laboratory animals. All procedures were performed in accordance to the National Institutes of Health *Guide for the care and use of laboratory animals,* and the Mexican Official Norm on the Principles of Laboratory Animal Care (NOM 062-ZOO-1999).

### Spinal cord injury

Adult female Sprague–Dawley rats weighing between 230 and 250 g were subjected to moderate SC contusion. Animals were anesthetized by an intramuscular injection of a mixture of ketamine (50 mg/kg, Probiomed, Mexico City, Mexico) and xylazine (10 mg/kg, Fort Dodge Laboratories, Fort Dodge, Iowa). Skin was opened in layers and a laminectomy was performed at the T9 level of the SC. Subsequently, a 10 g rod was dropped onto the SC from a height of 25 mm using the NYU impactor (NYU, New York). Functional recovery of all groups was assessed by the BBB locomotor scale [11, 12].

### Postoperative care

After SCI; animals were housed with food and water ad libitum, and received manual bladder voiding, three times a day for 2 weeks. To avoid infection, Enrofloxacin (Marvel, Mexico City, Mexico) was diluted into their drinking water at an approximate dose of 64 mg/kg/day for 1 week. Animals were carefully monitored for signs of infection, dehydration or auto mutilation with appropriate veterinary assistance as needed.

### Antigen (A91 peptide)

The A91 peptide was derived from the encephalitogenic sequence—amino acids 87-99—of the myelin basic protein (MBP). A non-encephalitogenic analogue was obtained by replacing lysine residue for alanine at position 91. The modified peptide was purchased from Invitrogen Life Technologies (San Diego CA, USA). Reverse-Phase HPLC confirmed the purity of the A91 peptide (>95%).

### Active immunization

Rats were immunized subcutaneously at the base of the tail with 200 μg of A91 in phosphate buffered saline (PBS), emulsified in an equal volume of complete Freund’s adjuvant (CFA) containing 0.5 mg/ml *Mycobacterium tuberculosis* (Sigma, St. Louis MO). Immunization was performed within a 60 min frame after injury.

### Removal and inhibition of scar formation

Two months after SCI, animals were anesthetized again as previously described. Thirty minutes after anesthetic induction, a longitudinal incision was performed until the fibrous tissue was identified. Using surgical microscopy, fibrosis was removed until meninges were clearly visible. A second longitudinal incision was then performed, and the meninges were referenced to the bordering muscles with a 9-0 suture. The exposed area was cleaned with saline solution, and necrotic tissue was eliminated. The scar from each stump was then removed through a single incision with a double-bladed scalpel. The surgeon was trained to perform reproducible procedures and was blinded to the group of animals.

This method is helpful to successfully remove the glial scar but also causes a slight lesion that allows a renewed production of growth factors, and thus the formation of a favorable microenvironment. It is important to mention that this type of lesion does not generate any additional neurological deficit. Once the scar was removed, its renewal was halted by adding α,α′-dipyridyl (DPY). DPY was injected directly –six times– into each stump of the SC by using a Hamilton syringe. Each injection deposited 2 µL volume of DPY (16 nmol) diluted in PBS.

### Functional recovery evaluation

Motor recovery was assessed by the BBB open-field locomotor scale method. Animals were evaluated weekly throughout 8 weeks by three observers blinded to the treatment. The average of the three scores was used.

### Semiquantitative gene expression

Gene expression of Tumor necrosis factor alpha (TNFα), Interferon gamma (INFγ), Interleukin 4 (IL-4), Transforming growth factor-beta 3 (TGFβ3), Brain derived neurotrophic factor (BDNF), Insulin-like growth factor-1 (IGF-1), Growth associated protein 43 (GAP-43), β-Actin, and HPRT [hypoxanthine phosphoribosyl transferase (housekeeping gene)] was determined by qRT-PCR, 120 days after injury. Total RNA (RNAt) was isolated from a 1.0 cm-long sample taken from the injury site of the spinal cord (0.5 cm caudal/0.5 cm rostral) using the Trizol method (Invitrogen, Carlsbad, CA, USA). Then, cDNA was synthesized from 2 µg of total RNA using the Superscript II transcriptase enzyme and Oligo dT (Invitrogen, Carlsbad, CA, USA). Primers were designed by Custom Primers OligoPerfect™ designer (http://tools.invitrogen.com) and confirmed by Primer analysis software [Oligo] (Molecular Biology Insights, Inc). The forward (F) and reverse (R) primers, amplicon size, and GeneBank entry numbers are listed in Table 1.

**Table 1:** Real-time PCR primers

Gene	GenBank	Primer sequence	Product length (bp)
TNFα	MN_012675	F tgacccccattactctgacc R ttcagcgtctcgtgtgtttc	152
INFϒ	MN_138880	F agcatggatgctatggaagg R ctgatggcctggttgtcttt	146
IL4	MN_201270	F gaaaaagggactccatgcac R tcttcaagcacggaggtaca	145
TGFβ3	NM_013174.1	F cccaacccccagctccaagcg R cagccactctgcggtggctc	132
BDNF	MN_001270630	F tggcctaacaatgtttgcag R cagctccacttagcctccac	114
IGF1	MN_001082477	F cttaggggctagcctcaggt R gttccgatgttttgcaggtt	158
GAP43	NM_017195.3	F ctaaggaaagtgcccgacag R gcaggagagacagggttcag	117
β-ACTIN	NM_007393	F agggccaaccgtgaaaag R gtggtacgaccagaggcatac	110
HPRT	NM_012583.2	F aagcttgctggtgaaaagga R caaagcctaaaagacagcgg	192

Reactions were performed with the FastStart Essential DNA Green Master kit (Roche, Diagnostics, Indianapolis, USA). The amplification was detected with a Light Cycler 96 instrument (Roche Diagnostics, Indianapolis, USA). All groups were compared to PBS-immunized animals. Relative concentrations were calculated by the Cq method (i.e., the cycle number in which the exponential amplification of the template begins) running the second derivative. The average value of each sample was obtained. Expression value from each analyzed gene was compared to that of the housekeeping gene by assigning a value equal to one to the latter for the normalization of the expression.

### Immunohistochemistry

Eight weeks after the therapeutic intervention, animals were euthanized with an overdose of sodium pentobarbital (80 mg/kg) and intracardiac perfusion with 4% paraformaldehyde. The affected portions of the SC were fixed overnight and then transferred to 30% sucrose for cryoprotection. Samples were embedded in Tissue-Tek (Miles Elkhart, IN, USA), and longitudinal frozen sections (40 μm thick) were performed. Immunohistochemical staining was carried out in order to count the amount of positive TH and 5-TH fibers. Tissues were incubated in 0.03% hydrogen peroxide to quench endogenous peroxidase activity. Subsequently, the tissue was incubated overnight with the following primary antibodies: monoclonal goat antibody against TH (1:2000; Chemicon), or polyclonal rabbit antibody against 5-HT (1:2000; Sigma-Aldrich). Following rinsing with PBS, samples were incubated for at least 2 h with donkey IgG anti-goat IgG (1:500; Chemicon) and Sheep IgG anti rabbit IgG (1:500; Abcam) secondary biotinylated antibodies. To visualize positive fibers, samples were incubated 5 min with Vector DAB kit (Vector laboratories, CA, USA). Then, samples were evaluated and analyzed by a blinded observer that counted individual fibers using a 20× objective (Olympus DP72, Japan). The amount of regenerating axons at the epicenter and 1 mm caudal to the lesion was assessed.

### Statistical analysis

Data is displayed as mean ± standard deviation (SD), and statistical significance was established when p ≤ 0.05. GraphPad Prism 5.0 (GraphPad Software, Inc. La Jolla, CA, USA) was employed in statistical analysis. Data from the assessment of functional recovery was analyzed using Kruskal–Wallis test, ANOVA with Bonferroni’s post hoc test for repeated measures, and Mann–Whitney U or Fisher’s exact probability tests. Gene expression results, as well as percentage of serotoninergic and catecholaminergic axon fibers, were analyzed by One-way ANOVA followed by Tukey–Kramer post hoc test.

## Results

### Immunization with neural derived peptides plus scar removal improved motor recovery after chronic SCI

**Fig. 1:**
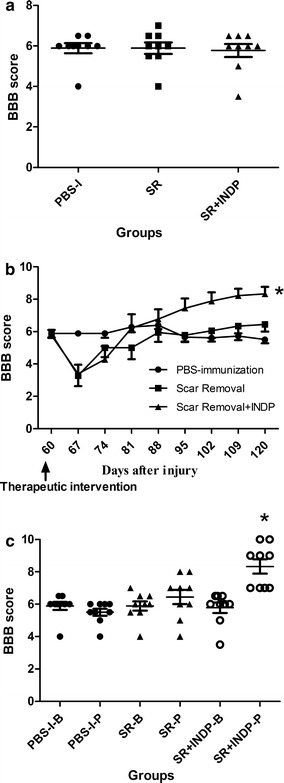
Locomotor recovery of rats before and after intervention. **a** There was not a significant difference among groups before intervention (p > 0.05; Kruskal–Wallis test. Mean ± SD is depicted for each group). **b** After intervention, a significantly better motor recovery was observed in the SR + INDP group. *p < 0.05, ANOVA for repeated measures with Bonferroni’s post hoc test. *Each point* represents the mean ± SD of 9 rats. **c** Comparison of the BBB score observed before (B) or after (P) therapeutic intervention. Rats subjected to SR + INDP presented a significant improvement after intervention. *p < 0.05 versus SR + INDP-B, Mann–Whitney U test. Mean ± SD is depicted for each group. *PBS-I* PBS-immunization, *SR* scar removal, *SR* + *INDP* scar removal + immunization with neural derived peptides

Evaluation of motor recovery before therapeutic intervention demonstrated that the BBB score was similar in the 3 groups (scar removal: 6.00 ± 1.031; PBS-immunization: 6.16 ± 0.25; scar removal + INDP: 6.33 ± 1.47; mean ± standard deviation (SD); Fig. 1a). Sixty days after intervention (120 days after SCI), rats submitted to scar removal + INDP showed a significant increase in motor recovery (8.11 ± 1.69; p < 0.05, ANOVA for repeated measures with Bonferroni’s post hoc test; Fig. 1b) when compared to those with scar removal alone (6.22 ± 1.85) or PBS-immunization (6.38 ± 0.48). Noteworthy, 55.5% of animals subjected to scar removal + INDP showed a locomotor improvement equal or above 9 in the BBB rating score (plantar paw placement with weight support instance). This percentage of animals was significantly different (p < 0.05; Fisher’s exact probability test) as compared to scar removal (0%) or PBS-immunized (0%) groups. When motor function (from each group) was compared before and after therapeutic intervention, we only found a significant difference in the scar removal + INDP group (p < 0.05; Wilcoxon test; Fig. 1c).

### Immunization with neural derived peptides plus scar removal generates a microenvironment where anti-inflammatory cytokine and regeneration-associated gene expression prevails

In order to confirm that scar removal + INDP indeed provides a permissive microenvironment for neural regeneration, inflammatory (TNFα and IFNγ) and anti-inflammatory (IL4, TGFβ), as well as regeneration-associated gene expression, was evaluated at the site of injury.

**Fig. 2:**
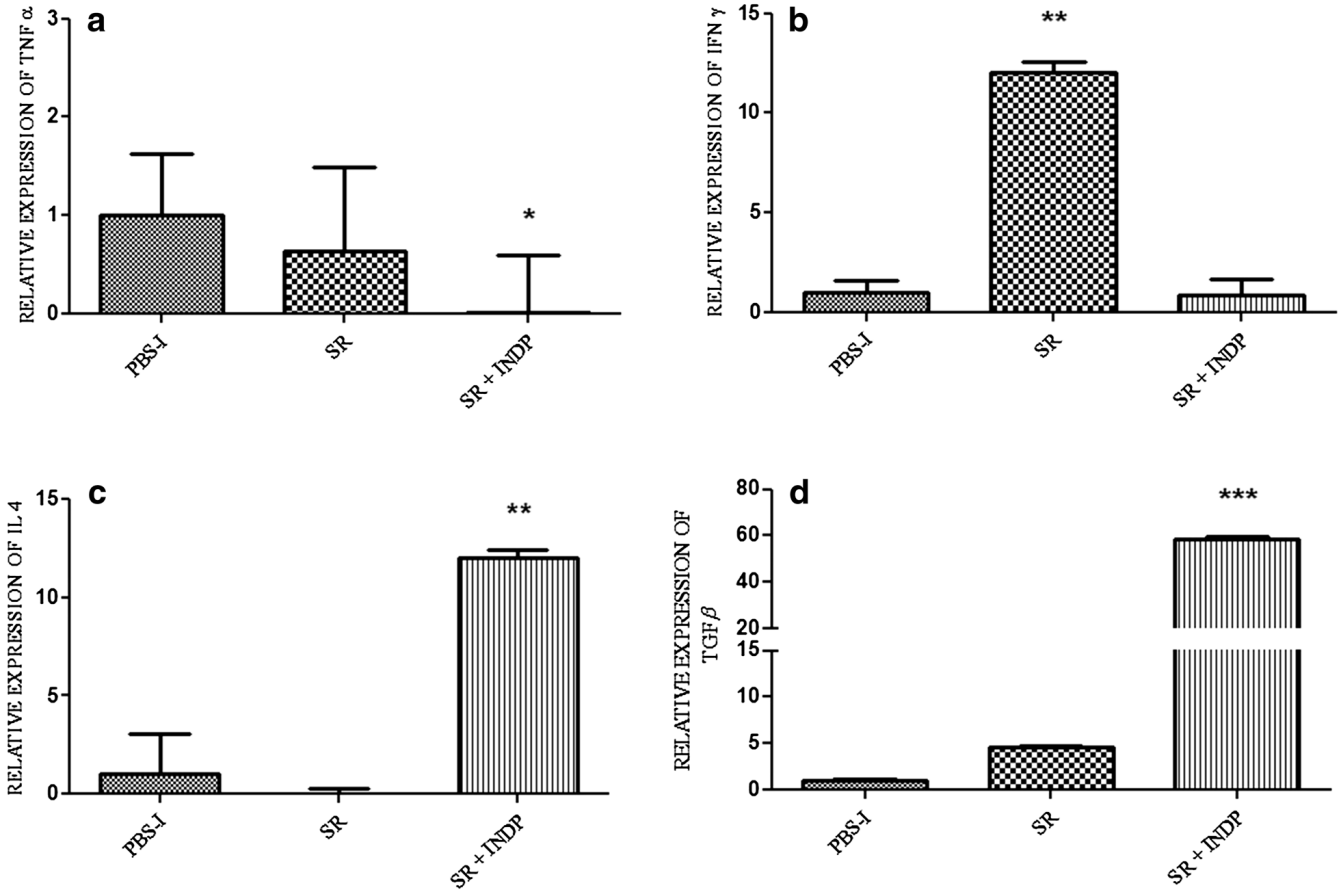
Relative expression of inflammation-related genes at the site of injury. The expression of TNFα (**a**) and INFγ (**b**) was significantly reduced in rats with scar removal + INDP. This group also presented a significant increase in IL4 (**c**) and TGFβ (**d**). *p < 0.05 versus all groups, **p < 0.001 versus all groups, ***p = 0.0001 versus all groups, One-way ANOVA followed by Tukey–Kramer post hoc analysis. *Each bar* represents the mean ± SD of 4 rats

Figure 2a shows that relative expression of TNFα was significantly reduced in rats with scar removal + INDP (0.02 ± 0.58) as compared to the one observed in the group of PBS-immunization (1.00 ± 0.62) or scar removal only (0.64 ± 0.84; p < 0.05, Kruskal–Wallis followed by Man-Whitney U test). The expression of the gene encoding for IFNγ was significantly increased in animals with scar removal alone (12.04 ± 0.55; p < 0.05 One-way ANOVA followed by Tukey test; Fig. 2b), but reduced in rats with scar removal + INDP (0.82 ± 0.83).

On the other hand, scar removal + INDP induced a significant increase of the genes encoding for IL4 (12.0 ± 0.34) and TGFβ (58.77 ± 0.59) when compared to PBS-immunized (1.00 ± 2.06; 1.00 ± 0.19, respectively) and scar removal groups (0.004 ± 0.26; 4.56 ± 0.09 respectively) (Fig. 2c, d; One-way ANOVA followed by Tukey–Kramer post hoc analysis).

**Fig. 3:**
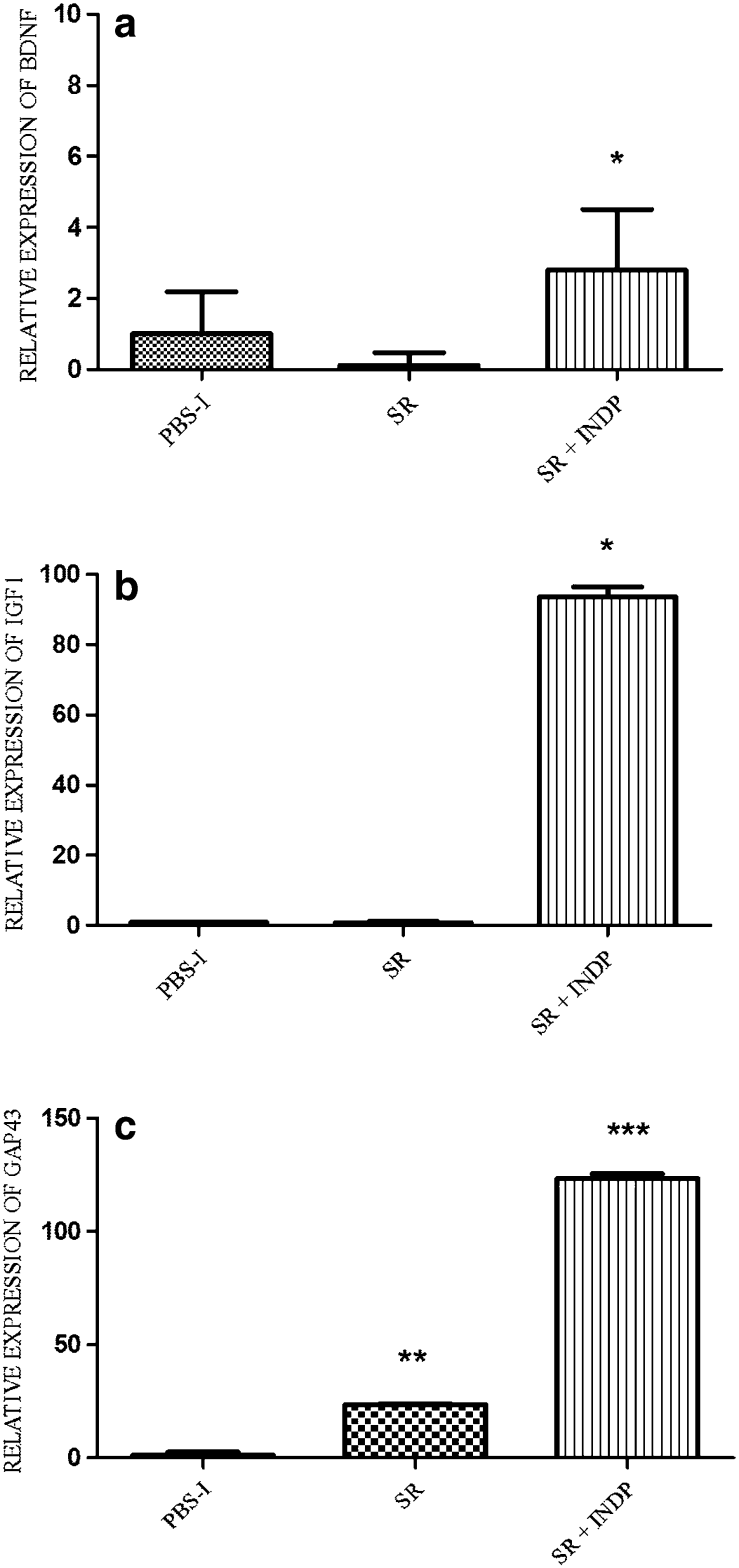
Relative expression of regeneration-associated genes at the site of injury. There was a significant increase in the expression of BDNF (**a**), IGF-1 (**b**) and GAP-43 (**c**) in the group with scar removal + INDP. *p < 0.05 versus all groups, **p < 0.05 versus PBS-I, ***p < 0.001 versus SR and p < 0.0001 versus PBS-I, One-way ANOVA followed by Tukey–Kramer post hoc test. *Each bar* represents the mean ± SD of 4 rats

Regeneration-associated genes (BDNF, IGF1, and GAP43) were also examined. The relative expression of BDNF (2.80 ± 1.71, p < 0.05 Kruskal–Wallis followed by Mann–Whitney U test), IGF1 (93.60 ± 0.74; p < 0.05 One-way ANOVA followed by Tukey–Kramer test), and GAP43 (123.22 ± 0.28; One-way ANOVA followed by Tukey test) was significantly increased in the group of rats with scar removal + INDP, when compared to PBS-immunized (1.00 ± 1.18; 1.00 ± 0.10; 1.00 ± 1.55, respectively) and scar removal (0.12 ± 0.35; 0.87 ± 0.51; 23.48 ± 0.33, respectively) groups (see Fig. 3a–c).

### Immunization with neural derived peptides plus scar removal promotes axonal regeneration

To determine if the permissive-anti-inflammatory microenvironment generated by scar removal + INDP had any positive effect on axonal regeneration; we assessed the percentage -obtained from the total number of fibers observed in sham-operated rats- of immunoreactive (IR) fibers to serotonin (5-HT) and tyrosine hydroxylase (TH) in the SC of studied animals.

**Fig. 4:**
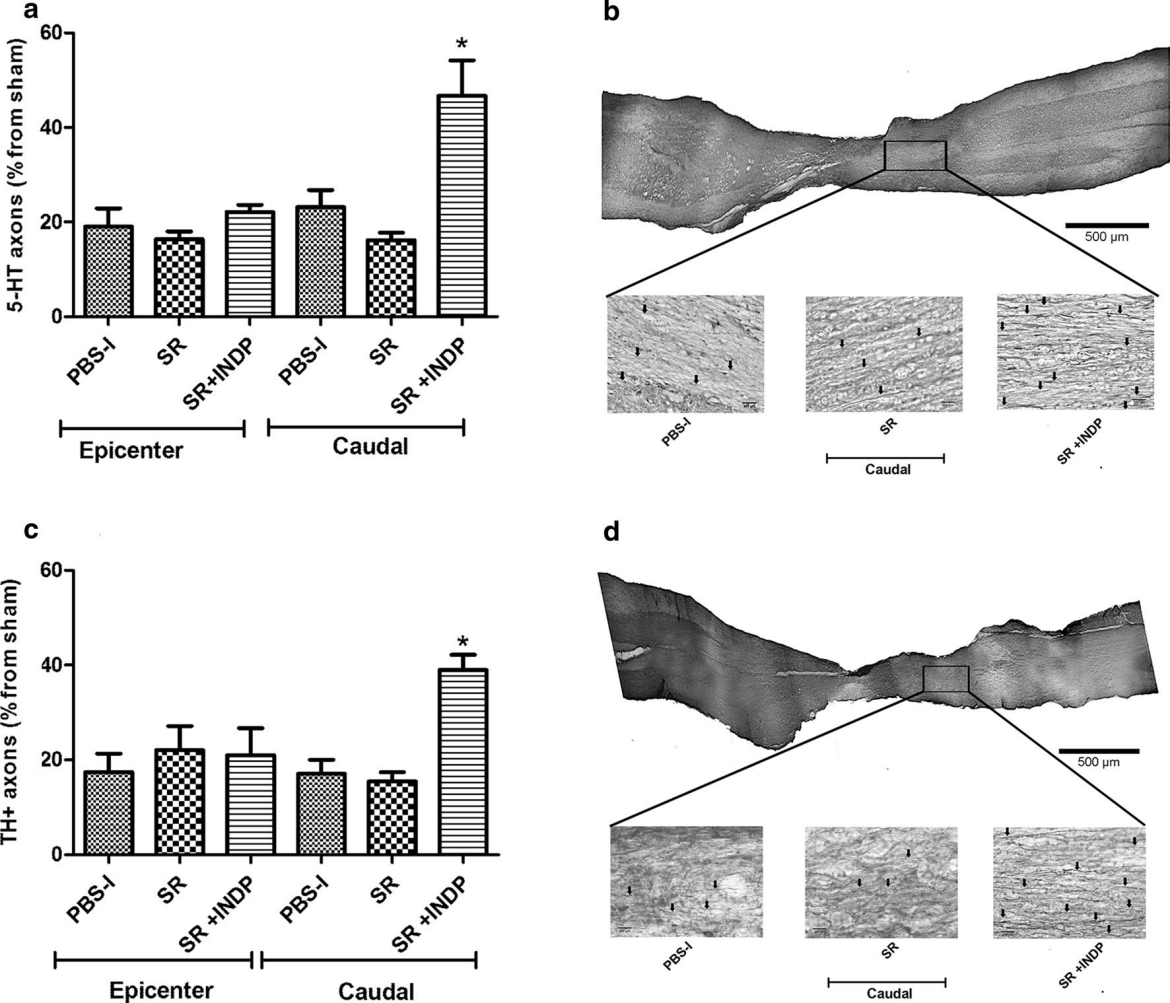
Percentage of axons observed at the epicenter and caudal stump of SCI rats after therapeutic intervention. The percentage was obtained from the total number of axons observed at the same level in sham-operated rats. Animals treated with scar removal + INDP (SR + INDP) presented a significant increase in the total amount of serotoninergic (**a**) and catecholaminergic (**b**) fibers. *p < 0.05, versus all groups, One-way ANOVA followed by Tukey–Kramer post hoc. *Each bar* represents the mean ± SD of 5 rats. *PBS-I* PBS immunization, *SR* scar removal. **c**, **d** Microphotographs representative of each analyzed therapy group

Figure 4a shows a significant increase of 5-HT-IR fibers in the caudal stump of rats subjected to scar removal + INDP. In this group, fiber percentage was significantly higher (46.70 ± 7.50; mean ± SD) than the one observed in PBS-immunized (23.18 ± 3.60) and scar removal (16.21 ± 2.0; p < 0.05, One-way ANOVA followed by Tukey–Kramer test) groups. Furthermore, scar removal + INDP induced a significant increase in the percentage of TH-IR axons in the caudal stump (39.0 ± 3.20; mean ± SD, p < 0.05, One-way ANOVA followed by Tukey–Kramer test; Fig. 4b) as compared to PBS-immunized (17.06 ± 2.97) or scar removed (15.49 ± 1.95) groups.

### Immunization with neural derived peptides alone also improves motor recovery in chronic SCI

**Fig. 5:**
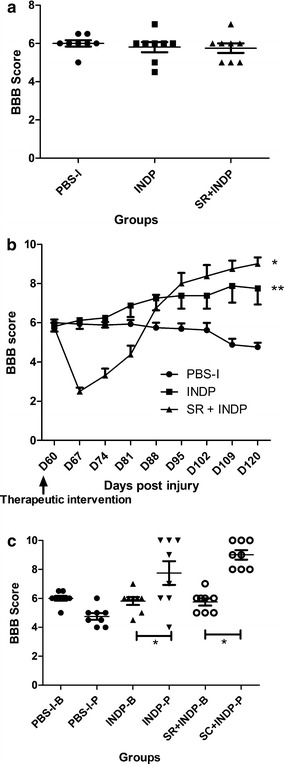
Motor recovery of rats subjected either to scar removal + INDP or INDP alone. **a** There was no significant difference among groups before intervention (p > 0.05; Kruskal–Wallis test. Mean ± SD is depicted for each group). **b** After intervention, rats treated with SR + INDP or INDP only showed a significant increase in motor performance when compared to PBS-immunized ones. *p < 0.01 versus PBS-I and p < 0.05 versus INDP, **p < 0.05 versus PBS-I. ANOVA for repeated measures with Bonferroni’s post hoc test. *Each point* represents the mean ± SD of 8 rats. **c** Comparison of the BBB score observed before (B) and after (P) therapeutic intervention. Rats subjected to combined SR + INDP or INDP alone presented significant improvement after intervention. *p < 0.05. Mann–Whitney U test. Mean ± SD is depicted for each group. *PBS-I* PBS-immunization, INDP immunization with neural derived peptide, *SR* + *INDP* scar removal + INDP

The second experiment was aimed at determining whether performing a slight injury was necessary in order to activate the beneficial effect of PA, or whether INDP alone was sufficient to promote neural restoration in the chronic phase of SCI. The study population for this experiment consisted of twenty-four rats subjected to SCI. Two months after injury, rats were allocated into three groups as follows: (1) sham operated rats (SCI with surgical intervention but no scar removal) immunized with PBS (n = 8); (2) rats with scar removal + INDP (n = 8); (3) rats with INDP plus sham operation with no scar removal (n = 8). Rats treated with INDP alone showed improved locomotion recovery, although the effect was not as marked as the one observed in the combined INDP + scar removal group (Fig. 5).

Baseline BBB scores calculated 60 days after SCI were similar among all groups (5.8 ± 0.1; Fig. 5a). At the end of the follow-up period after intervention, animals with scar removal + INDP presented higher motor recovery (9.0 ± 0.3; Fig. 5b) than those with INDP alone (7.8 ± 0.8) or those in the PBS-immunized group (5.75 ± 0.2). In the same way, the group receiving combined scar removal + INDP presented the highest percentage of rats with BBB scores equal to or above 9 points (62.5%) when compared to those with INDP alone (50%) and PBS-immunization (0%). The difference between the combined INDP + scar removal group and the INDP alone group was statistically significant (p < 0.05; Fisher’s exact probability test), as well as between these two groups and the PBS-immunized group (p < 0.05). Comparison of motor function before and after therapeutic intervention showed that both scar removal + INDP and INDP alone groups presented significant recovery (p < 0.05; Wilcoxon test; Fig. 5c).

### The microenvironment generated by INDP alone is also permissive, although to a lesser degree than the one induced by scar removal + INDP

**Fig. 6:**
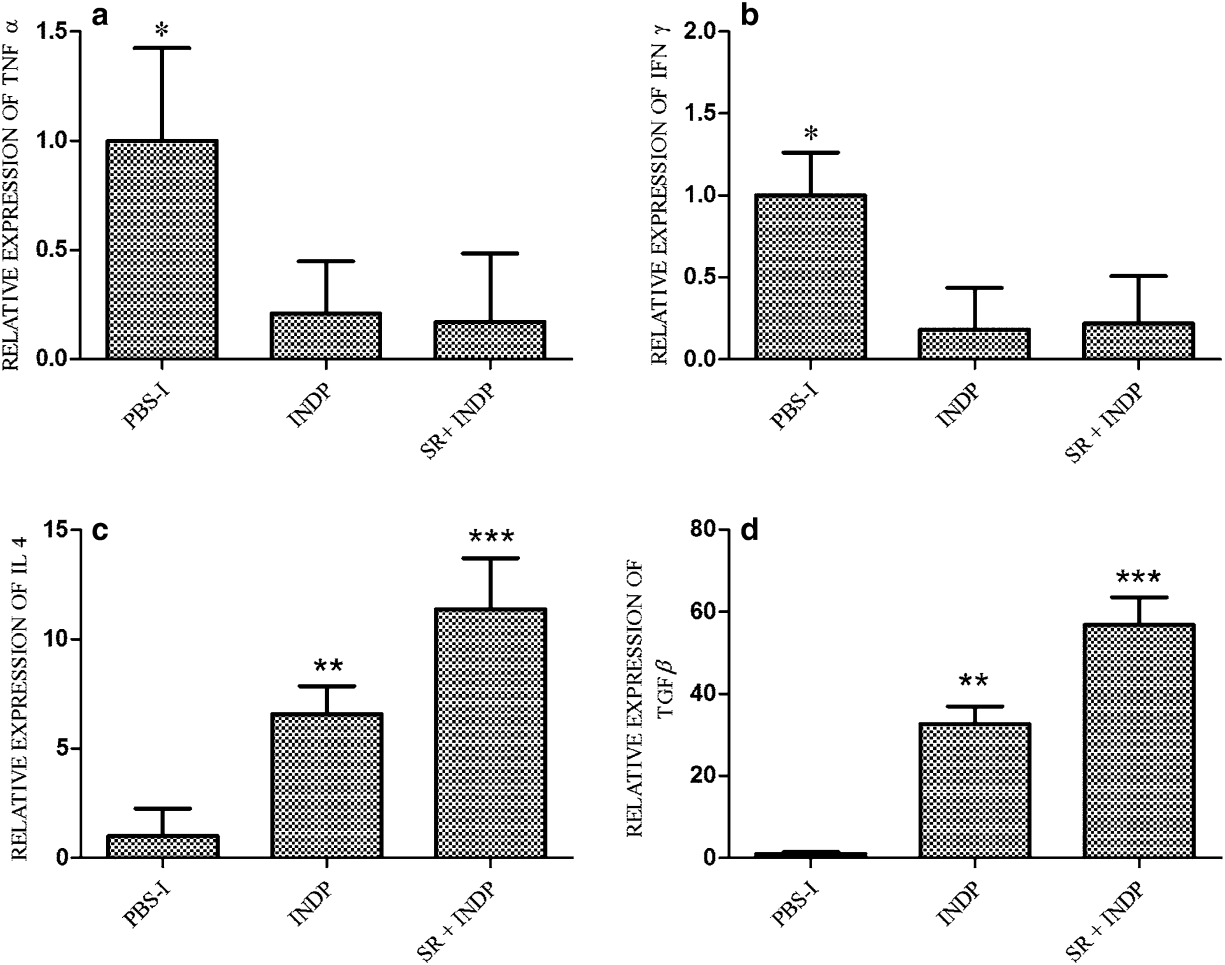
Inflammation-related gene expression in INDP-treated rats at the site of injury. TNFα (**a**) and INFγ (**b**) expression was significantly reduced in the scar removal + INDP treatment group and the INDP alone group. Both groups also presented a significant increase in IL4 (**c**) and TGFβ (**d**) expression. *p < 0.05 versus all groups, **p < 0.05 versus PBS-I, ***p = 0.001 versus PBS-I and p < 0.05 versus INDP, One-way ANOVA followed by Tukey–Kramer post hoc analysis. *Each bar* represents the mean ± SD of 4 rats

**Fig. 7:**
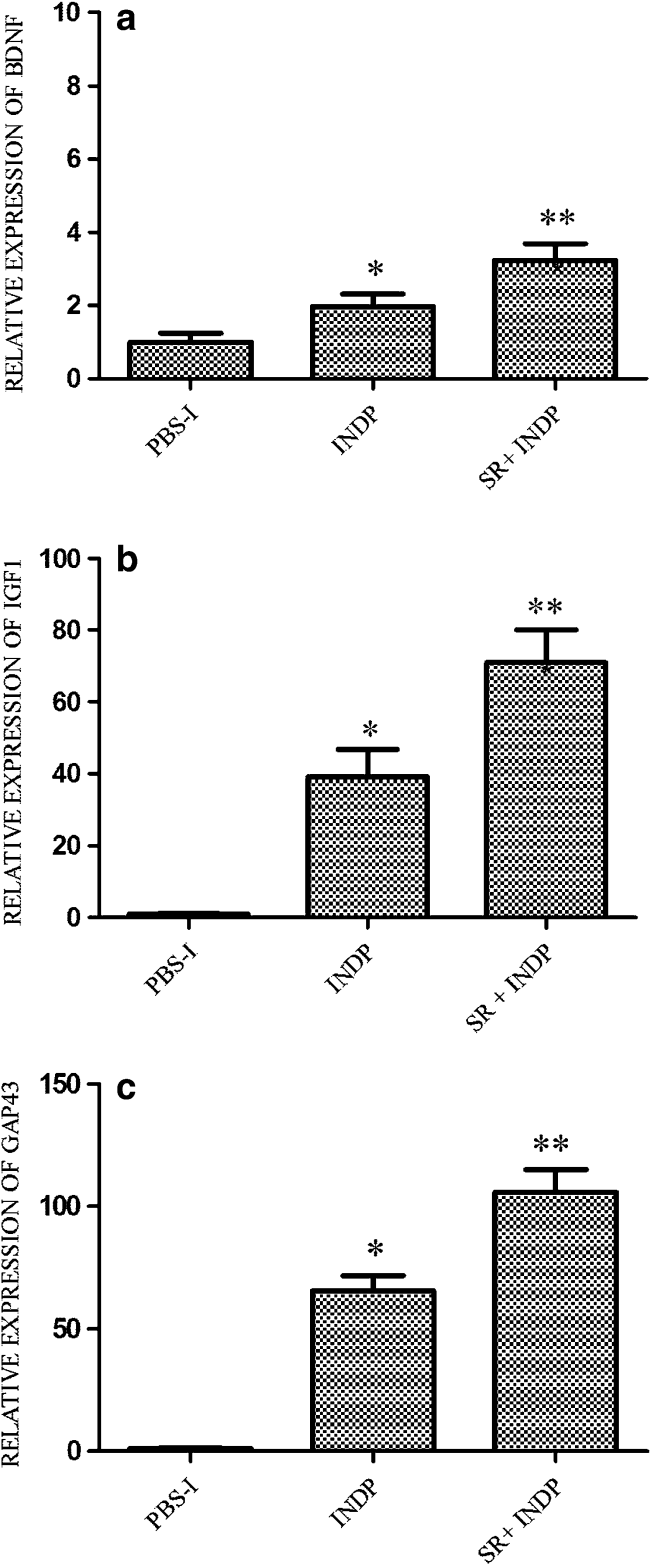
Regeneration-associated genes in INDP-treated rats at the site of injury. There was a significant increase in BDNF (**a**), IGF-1 (**b**) and GAP-43 (**c**) expression in rats treated with scar removal + INDP and those with only INDP. *p < 0.05 versus PBS-I, **p < 0.01 versus PBS-I and p < 0.05 versus INDP, One-way ANOVA followed by Tukey–Kramer post hoc test. *Each bar* represents the mean ± SD of 4 rats

IFNγ and TNFα gene expression was significantly reduced in INDP treated rats when compared to PBS-immunized ones (0.18 ± 2 and 0.21 ± 2 respectively; p < 0.05, One-way ANOVA followed by Tukey–Kramer post hoc test). In a similar manner, there was a reduction in the expression of these genes in animals with scar removal + INDP (0.21 ± 3 and 0.17 ± 2 respectively; Fig. 6a, b, p < 0.05 vs. PBS-immunized group). There was no significant difference between INDP alone and scar removal + INDP (p > 0.05). IL-4 and TGFβ gene expression was significantly increased in rats treated only with INDP versus PBS-immunized rats (6.6 ± 1 and 32.6 ± 4 respectively; p < 0.05, One-way ANOVA followed by Tukey–Kramer post hoc test), although results were not as high as those observed in the scar removal + INDP group (11.4 ± 2 and 56.8 ± 7 respectively; p < 0.01 vs. PBS-immunized rats and p < 0.05 vs. INDP alone; Fig. 6c, d). Expression of regeneration-associated genes was significantly increased in rats treated with INDP alone (see Fig. 7; BDNF: 1.98 ± 0.3; IGF1: 39.2 ± 8; GAP43: 65.8 ± 6); however, it was also lower than that of the scar removal + INDP group (BDNF: 3.2 ± 0.4; IGF1: 70.9 ± 0.9; GAP43: 105.8 ± 9, p < 0.05 One-way ANOVA followed by Tukey test).

**Fig. 8:**
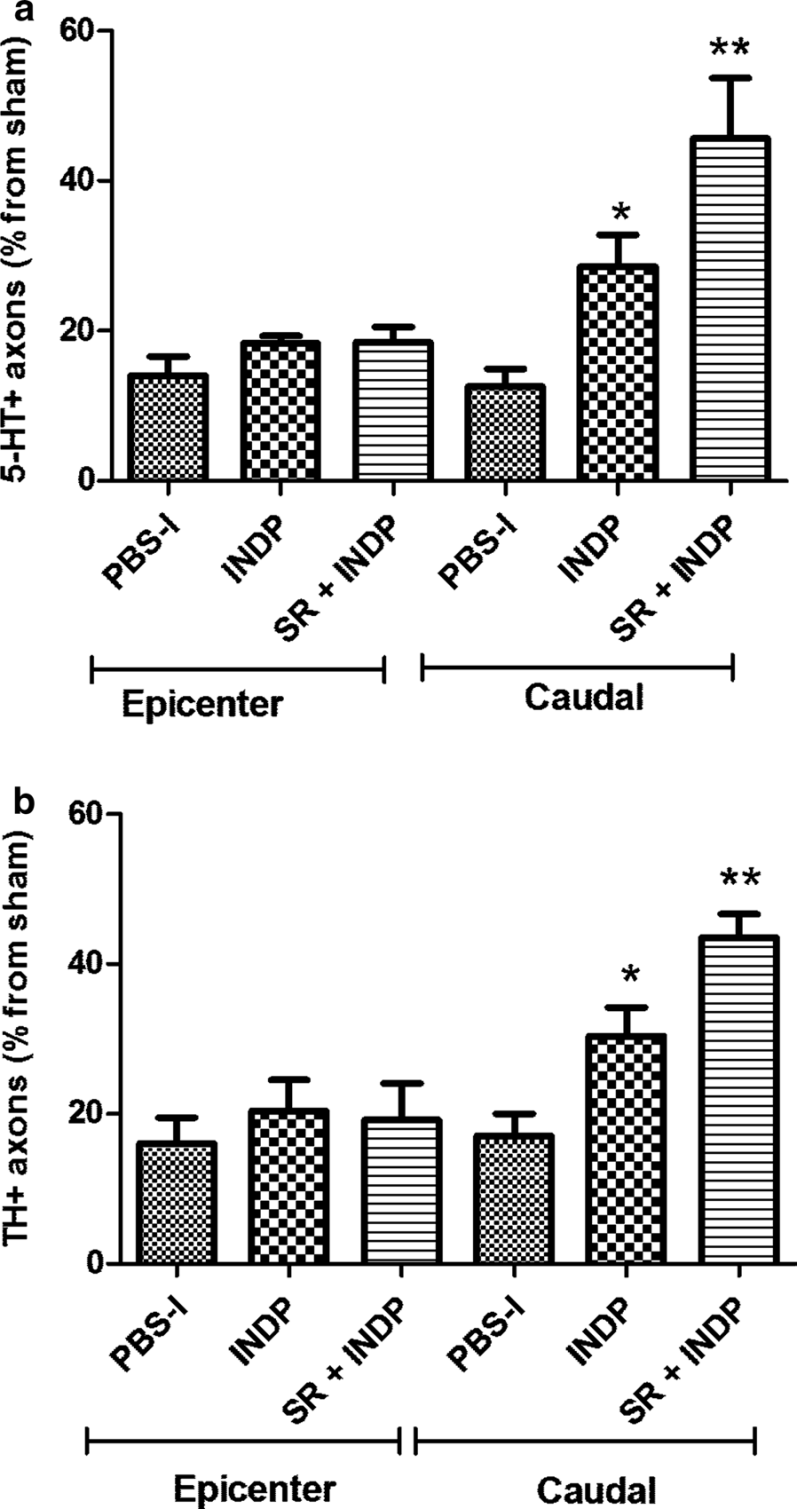
Percentage of axons observed at the epicenter and caudal stump of SCI rats after therapeutic intervention. The percentage was obtained from the total number of axons found at the same level in sham-operated rats. Animals treated either with scar removal + INDP (SR + INDP), or INDP alone presented a significant increase in the percentage of serotoninergic (**a**) and catecholaminergic (**b**) fibers. *p < 0.05, versus PBS-I, **p < 0.01 versus PBS-I, and p < 0.05 versus INDP. One-way ANOVA followed by Tukey–Kramer post hoc. *Each bar* represents the mean ± SD of 4 rats. *PBS-I* PBS immunization, *INDP* immunization with neural derived peptide

Figure 8a shows a significant increase of 5-HT-IR fibers in the caudal stump of rats subjected to INDP alone. Fiber percentage in this group was significantly higher (28.64 ± 4.20; mean ± SD) than the one observed in PBS-immunized (12.58 ± 2.38) rats (p < 0.05, One-way ANOVA followed by Tukey–Kramer test). Nevertheless, the percentage of axons presented by the INDP-treated group was lower than the one observed in scar removal + INDP-treated animals (45.61 ± 8.0). There was a significant increase in the percentage of TH-IR axons in the caudal stump in the combined treatment group (43.53 ± 3.1; mean ± SD, p < 0.05, One-way ANOVA followed by Tukey–Kramer test; Fig. 8b) when compared to INDP (30.39 ± 3.79) or PBS-immunized (17.06 ± 2.97) groups.

## Discussion

Previous studies have demonstrated that INDP promotes neuroprotection after CNS damage [13, 14]. Moreover, recent investigations have also shown that this strategy is capable of inducing neural restoration [15]. These beneficial effects are observed especially when INDP is administered immediately after injury. In the present study, we explored the effect of this therapy in the chronic phase of SCI, a scenario characterized by a marked period of stability where protective and restorative promoters are no longer present. During this phase, the elements of the immune system are also under diminished activity; even though, they continue with their remodeling labor [4], its grade of activation is low. In order to break with this unproductive phase and simultaneously eliminate the physical barrier presented by gliosis and collagen fibers, we designed our first experiment to include scar removal and modulation of the immune response by means of INDP. We envisioned that scar removal could provide a favorable environment to obtain the beneficial effects of INDP. Under the light of this contention, the present work had the objective to elucidate the effect of this combination treatment in chronic SCI. Collectively, our results showed that scar removal + INDP, induced a significant improvement in motor recovery. This beneficial effect was accompanied by a compelling increase in the expression of anti-inflammatory (IL-4 and TGFβ) and regeneration-associated genes (BDNF, IGF1, and GAP-43). In the same way, this group of rats exhibited a better number of regenerating axons. These results could be of remarkable significance since they reveal that stability and progressive degeneration observed at the chronic phase of SCI, may be interrupted and moreover, it could be reverted into a suitable microenvironment for neurological recovery.

Throughout the second experiment, we demonstrated that treatment with INDP alone also has the ability to revert the state of stability and progressive degeneration observed in the chronic phase of injury. However, rats who received this therapy did not experience the same degree of benefits as those in the scar removal + INDP group.

A number of diverse factors could be responsible for the difference observed between these two groups. First among these, -as aforementioned- is the establishment of a permissive microenvironment for increased INDP activity through scar removal. In contrast, the lack of scar removal hinders the ability of INDP to exert its beneficial effects, due to a less permissive microenvironment. Another possible factor contributing to the lower effect observed in rats treated with INDP alone is the physical and chemical barrier posed by the scarring itself. In this regard, conflicting evidence exists over the importance of scar tissue as a barrier for axonal growth. A number of reports supporting the role of scar tissue as a barrier have shown that its removal facilitates neural restoration [16–18]. However, recent studies have also suggested that scar tissue is not necessarily an obstacle for regeneration and may even be necessary for neural restoration [19]. In the present work, we demonstrated that INDP alone promotes motor recovery, although this effect is augmented with glial scar removal. Further detailed studies evaluating the necessity of scar removal in INDP treatment should be carried out.

In the present study, over 50% of rats treated either with scar removal + INDP or INDP alone presented an improvement in locomotion, scoring equal or higher than 9 in the BBB rating scale (plantar paw placement with weight support in stance). Interestingly, these groups showed a BBB score of 6 before treatment (60 days after SCI). This encouraging effect could only be the result of a regenerative process, since, after therapy some animals recovered not only the capacity to support their weight, but regain the ability to perform plantar stepping (BBB score of 10). Since these findings suggested the appearance of a regenerative process as the responsible for generating the motor improvement, we investigated whether the microenvironment generated in each group (scar removal +INDP and INDP alone) was propitious for inducing this favorable outcome. In this way, we found a significant amount of IL-4 and TGFβ, which are strongly related to neuroprotective and regenerative processes. For instance, IL-4 may exert neuroprotective effects by regulating the acute and chronic macrophage responses [20]. In the same way, this cytokine promotes growth, phagocytic activity, and proliferation of microglial cells. IL-4 also inhibits the production of nitric oxide (NO) and pro-inflammatory cytokines such as TNFα and INFγ [21].

On the other hand, IL-4 also provides beneficial effects on neural restoration. It has been shown that this cytokine is capable of inducing axonal outgrowth in an ex vivo model, as incubation of neuronal cultures with IL-4 promoted an enhanced elongation of axons. This study demonstrated the recovery of injured neurons by activation of neuronal IL-4 receptors, enhancing neurotrophin signaling via the AKT and MAPK pathways [22]. Moreover, it has been demonstrated that IL-4 increases the expression of IGF-1, an important molecule that contributes to neurite outgrowth [23]. Our work showed that INDP induced a favorable IL-4 microenvironment; thereby, we could expect its beneficial actions at the site of injury.

TGFβ could also be supporting restorative mechanisms after SCI, since it participates in the regulation of neuron survival and orchestration of repair processes in the CNS [24]. TGFβ is a pleiotropic molecule with specific key roles in cell differentiation, proliferation, migration, extracellular matrix metabolism, and immunosuppression [24–27]. Evidence suggests that this molecule could also play a role in the regulation of adult neurogenesis [27].

The increment of IL-4 and TGFβ, as well as the reduction of TNFα and INFγ, contribute to the induction of a permissive microenvironment that is favorable for the action of restorative molecules. With this in regard, studies in our laboratory have already shown that INDP promotes an in vitro and in vivo production of neurotrophic factors [13, 15]. Interestingly, it has been demonstrated that the production of this molecules could last until chronic stages of SCI [28]. In the present study, we observed a significant increase of regeneration-associated genes such as BDNF, IGF-1, and GAP-43 in rats with chronic SCI treated with scar removal + INDP or INDP alone. This finding could explain, at least in part, the motor recovery observed in both of these groups, with animals presenting a significant increase in the expression of genes encoding for molecules intimately related to neural restoration.

BDNF also plays a significant role in neural repair and plasticity, as it exerts different effects after trauma to the CNS. Some of these effects include neurogenesis, axonal sprouting, myelination, and adaptive synaptic plasticity [29, 30]. In fact, it is endowed by immediate actions that have a direct impact on synaptic transmission [31]. On the other hand, IGF-1 promotes neurite outgrowth of various neuronal populations both in vitro and in vivo [32]. Moreover, expression of IGF-1R mRNA promotes spinal motor neuron survival and enhances neurite outgrowth in sympathetic neurons [31]. The increased release of BDNF and IGF-1 by microglia and macrophages has been associated with an increased neurogenesis from endogenous neural precursor cells after SCI. Moreover, BDNF may be coupled to the induction of GAP-43 [33, 34], a common mediator of the regenerative effect of BDNF [35]. Interestingly, GAP-43 is essential for the neurotrophic functions of BDNF [33], in fact; in models of cervical axotomy, BDNF injection stimulates GAP-43 expression, and consequently induces axogenesis and repair [36]. These findings could explain the increase of GAP-43 observed in the group of rats treated with combined scar removal + INDP and in those receiving INDP alone. GAP-43 is involved in translating fundamental signals for growth cone guidance [37]. Also, several studies point to a possible role for GAP-43 in regulating neurotransmitter release [33, 38, 39]. GAP-43 is a useful marker, and plays a major role in neurite formation, regeneration and neuroplasticity [34]. Altogether, these findings support the idea that BDNF, IGF-1, and GAP-43 strongly contribute to neural restoration. In the present investigation, the increase in gene expression encoding for these molecules was associated to a significant augmentation in the number of descending fibers at the caudal stump of the SC.

Serotonergic (5-HT-positive) and catecholaminergic (TH-positive) axons in the SC are descending neuron fibers located in the raphe and coeruleus nucleus respectively. SCI produces a decrease in these fibers at caudal levels from the site of injury [40, 41]. The microenvironment observed in rats treated with scar removal + INDP and in those treated exclusively with INDP was associated with a significant increase in the number of fibers at the caudal segment of the SC. This finding supports the idea of a permissive microenvironment that promotes the formation of new fibers.

Finally, this study also showed an increased expression of the gene encoding for INFγ in rats subjected only to scar removal. This observation provides evidence regarding the modulatory effect exerted by INDP (in the case of rats treated either with scar removal + INDP or with INDP alone). Furthermore, these results emphasize that even after minimum injury—scar removal—there is a significant inflammatory response that could be playing an important role in tissue degeneration, and also over the lack of functional regeneration. With this in regard, there is evidence demonstrating that pro-inflammatory stimuli suppress the production of neural growth factors [42]. This finding was partially supported by our results since animals treated only with scar removal, did no present a significant increase in the expression of the genes encoding for BDNF or IGF-1. Interestingly, scar removal induced a significant increase in the expression of the gene encoding for GAP-43. However, none of the animals reached the score of 9 on the BBB scale. The latter demonstrates, in some way, that the microenvironment induced by INDP is required to achieve the best conditions as to gain a better neurological improvement.

## Conclusions

Collectively, our results suggest that both, combined therapy consisting of scar removal + INDP and INDP alone could substantially modify the non-permissive microenvironment prevailing at the chronic phase of SCI, providing the opportunity to promote a higher motor recovery. Of these, combined scar removal + INDP therapy demonstrated greater beneficial effects.

## Abbreviations

INDP: immunization with neural derived peptides
SCI: spinal cord injury
TI: therapeutic intervention
TH: tyrosine hydroxylase
5-HT: 5-hydroxytryptamine
SC: spinal cord
CNS: central nervous system
PA: protective autoimmunity
MBP: myelin basic protein
BBB: Basso, Beattie and Bresnahan
CFA: complete Freund’s adjuvant
DPY: α,α′-dipyridyl
TNFα: tumor necrosis factor alpha
INFγ: interferon gamma
IL-4: interleukin 4
TGFβ3: transforming growth factor-beta 3
BDNF: brain derived neurotrophic factor
IGF-1: insulin-like growth factor-1
GAP-43: growth associated protein 43
HPRT: hypoxanthine phosphoribosyl transferase
SD: standard deviation
IR: immuno reactive
NO: nitric oxide
PBS: phosphate buffered saline
PBSI: PBS immunization
SR: scar removal

## References

[1] Lemke M, Demediuk P, McIntosh TK, Vink R, Faden AI (1987). Alterations in tissue Mg++, Na+ and spinal cord edema following impact trauma in rats. Biochem Biophys Res Commun.

[2] Akdemir O, Uçankale M, Karaoğlan A, Barut S, Sağmanligil A, Bilguvar K (2008). Therapeutic efficacy of SJA6017, a calpain inhibitor, in rat spinal cord injury. J Clin Neurosci.

[3] Zhao RR, Fawcett JW (2013). Combination treatment with chondroitinase ABC in spinal cord injury-breaking the barrier. Neurosci Bull.

[4] Beck KD, Nguyen HX, Galvan MD, Salazar DL, Woodruff TM, Anderson AJ (2010). Quantitative analysis of cellular inflammation after traumatic spinal cord injury: evidence for a multiphasic inflammatory response in the acute to chronic environment. Brain.

[5] Ibarra A, Hauben E, Butovsky O, Schwartz M (2004). The therapeutic window after spinal cord injury can accommodate T cell-based vaccination and methylprednisolone in rats. Eur J Neurosci.

[6] Schwartz M, Baruch K (2014). The resolution of neuroinflammation in neurodegeneration: leukocyte recruitment via the choroid plexus. EMBO J.

[7] Schwartz M (2003). Control of microglial activity by protective autoimmunity. Adv Mol Cell Biol.

[8] Ibarra A, García E, Flores N, Martiñón S, Reyes R, Campos MG (2010). Immunization with neural-derived antigens inhibits lipid peroxidation after spinal cord injury. Neurosci Lett.

[9] Bravo G, Ibarra A, Guizar-Sahagún G, Rojas G, Hong E (2007). Indorenate improves motor function in rats with chronic spinal cord injury. Basic Clin Pharmacol Toxicol.

[10] Ziv Y, Schwartz M (2008). Orchestrating brain-cell renewal: the role of immune cells in adult neurogenesis in health and disease. Trends Mol Med.

[11] Basso DM, Beattie MS, Bresnahan JC (1996). Graded histological and locomotor outcomes after spinal cord contusion using the NYU weight-drop device versus transection. Exp Neurol.

[12] Hauben E, Ibarra A, Mizrahi T, Barouch R, Agranov E, Schwartz M (2001). Vaccination with a Nogo-A-derived peptide after incomplete spinal-cord injury promotes recovery via a T-cell-mediated neuroprotective response: comparison with other myelin antigens. Proc Natl Acad Sci USA.

[13] Martiñon S, García E, Flores N, Gonzalez I, Ortega T, Buenrostro M (2007). Vaccination with a neural-derived peptide plus administration of glutathione improves the performance of paraplegic rats. Eur J Neurosci.

[14] Ibarra A, Avendaño H, Cruz Y (2007). Copolymer-1 (Cop-1) improves neurological recovery after middle cerebral artery occlusion in rats. Neurosci Lett.

[15] Cruz Y, Lorea J, Mestre H, Kim-Lee JH, Herrera J, Mellado R (2015). Copolymer-1 promotes neurogenesis and improves functional recovery after acute ischemic stroke in rats. PLoS ONE.

[16] Jefferson S, Tester N, Howland D (2011). Chondroitinase ABC Promotes Recovery of adaptive limb movements and enhances axonal growth caudal to spinal hemisection. J Neurosci.

[17] Zhang S, Kluge B, Huang F, Nordstrom T, Doolen S, Gross M (2007). Photochemical scar ablation in chronically contused spinal cord of the rat. J Neurotrauma.

[18] Goncalves MB, Malmgvist T, Clarke E, Hubens CJ, Grist J, Hobbs C (2015). Neuronal RARβ signalling modulates PTEN activity directly in neurons and via exosome transfer in astrocytes to prevent glial scar formation and induce spinal cord regeneration. J Neurosci.

[19] Sekiya T, Holley MC, Hashido K, Ono K, Shimomura K, Horie RT (2015). Cells transplanted onto the surface of the glial scare reveal hidden potential for functional regeneration. Proc Natl Acad Sci.

[20] Lee SI, Jeong SR, Kang YM, Han DH, Jin BK, Namgung U (2010). Endogenous expression of interleukin-4 regulates macrophage activation and confines cavity formation after traumatic spinal cord injury. J Neurosci Res.

[21] Vidal PM, Lemmens E, Dooley D, Hendrix S (2013). The role of “anti-inflammatory” cytokines in axon regeneration. Cytokine Growth Factor Rev.

[22] Walsh JT, Hendrix S, Boato F, Smirnov I, Zheng J, Lukens JR (2014). MHCII-independent CD4+ T cells protect injured CNS neurons via IL-4. J Clin Invest.

[23] Butovsky O, Ziv Y, Schwartz A, Landa G, Talpalar AE, Pluchino S (2006). Microglia activated by IL-4 or IFN-gamma differentially induce neurogenesis and oligodendrogenesis from adult stem/progenitor cells. Mol Cell Neurosci.

[24] Krieglstein K, Strelau J, Schober A, Sullivan A, Unsicker K (2002). TGF-beta and the regulation of neuron survival and death. J Physiol Paris.

[25] Matejuk A, Dwyer J, Hopke C, Vandenbark AA, Offner H (2004). Opposing roles for TGF-β1 and TGFβ3 isoforms in experimental autoimmune encephalomyelitis. Cytokine.

[26] Heupel K, Sargsyan V, Plomp JJ, Rickmann M, Varoqueaux F, Zhang W (2008). Loss of transforming growth factor-beta 2 leads to impairment of central synapse function. Neural Dev.

[27] Dobolyi A, Vincze C, Pál G, Lovas G (2012). The neuroprotective functions of transforming growth factor beta proteins. Int J Mol Sci.

[28] Martiñon S, Toscano-Tejeida D, Garcia-Vences E, Flores-Romero A, Rodriguez-Barrera R, Ferrusquia M (2016). Long-term production of BDNF and NT-3 induced by A91-immunization after spinal cord injury. BMC Neurosci.

[29] Weishaupt N, Blesch A, Fouad K (2012). BDNF: the career of a multifaceted neurotrophin in spinal cord injury. Exp Neurol.

[30] Harvey AR, Lovett SJ, Majda BT, Yoon JH, Wheeler LPG, Hodgetts SI (2014). Neurotrophic factors for spinal cord repair: which, where, how and when to apply, and for what period of time?. Brain Res.

[31] Kovalchuk Y, Holthoff K, Konnerth A (2004). Neurotrophin action on a rapid timescale. Curr Opin Neurobiol.

[32] Bondy CA (1991). Transient IGF-I gene expression during the maturation of functionally related central projection neurons. J Neurosci.

[33] Gupta SK, Mishra R, Kusum S, Spedding M, Meiri KF, Gressens P (2009). GAP-43 is essential for the neurotrophic effects of BDNF and positive AMPA receptor modulator S18986. Cell Death Differ.

[34] Wei HF, Zeng BF, Chen YF, Chen L, Gu YD (2011). BDNF and GAP43 contribute to dynamic transhemispheric functional reorganization in rat brain after contralateral C7 root transfer following brachial plexus avulsion injuries. Neurosci Lett.

[35] Madinier A, Bertrand N, Mossiat C, Prigent-Tessier A, Beley MC, Garnier P (2009). Microglial involvement in neuroplastic changes following focal brain ischemia in rats. PLoS ONE.

[36] Kobayashi NR, Fan DP, Giehl KM, Bedard AM, Wiegand SJ, Tetzlaff W (1997). BDNF and NT-4/5 prevent atrophy of rat rubrospinal neurons after cervical axotomy, stimulate GAP-43 and Talpha1-tubulin mRNA expression, and promote axonal regeneration. J Neurosci.

[37] Frey D, Laux T, Xu L, Schneider C, Caroni P (2000). Shared and unique roles of CAP23 and GAP43 in actin regulation, neurite outgrowth, and anatomical plasticity. J Cell Biol.

[38] Benowitz LI, Perrone-Bizzozero NI (1991). The relationship of GAP-43 to the development and plasticity of synaptic connections. Ann N Y Acad Sci.

[39] Donovan SL, Mamounas LA, Andrews AM, Blue ME, McCasland JS (2002). GAP-43 is critical for normal development of the serotonergic innervation in forebrain. J Neurosci.

[40] Anderson EG, Davidoff RS (1983). The serotonin system of the spinal cord. Handbook of the spinal cord.

[41] Saruhashi Y, Young W, Perkins R (1996). The recovery of 5-HT immunoreactivity in lumbosacral spinal cord and locomotor function after thoracic hemisection. Exp Neurol.

[42] Lees JR (2015). Cytokine Interferon gamma in autoimmunity: a complicated player on a complex stage. Cytokine.

